# Elucidation of the Role of Lipids in Late Endosomes on the Aggregation of Insulin

**DOI:** 10.1021/acschemneuro.3c00475

**Published:** 2023-09-08

**Authors:** Ritu Joshi, Kiryl Zhaliazka, Aidan P. Holman, Dmitry Kurouski

**Affiliations:** † Department of Biochemistry and Biophysics, Texas A&M University, College Station, Texas 77843, United States; ‡ Department of Entomology, Texas A&M University, College Station, Texas 77843, United States; § Department of Biomedical Engineering, Texas A&M University, College Station, Texas 77843, United States

**Keywords:** insulin, bis(monoacylglycero)phosphate, phosphatidylcholine, AFM, LDH, fibrils

## Abstract

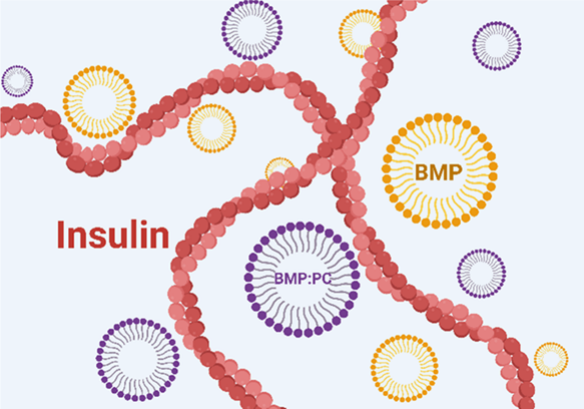

Abrupt aggregation of misfolded proteins is the underlying molecular cause of numerous pathologies including diabetes type 2 and injection amyloidosis. Although the exact cause of this process is unclear, a growing body of evidence suggests that protein aggregation is linked to a high protein concentration and the presence of lipid membranes. Endosomes are cell organelles that often possess high concentrations of proteins due to their uptake from the extracellular space. However, the role of endosomes in amyloid pathologies remains unclear. In this study, we used a set of biophysical methods to determine the role of bis(monoacylglycero)phosphate (BMP), the major lipid constituent of late endosomes on the aggregation properties of insulin. We found that both saturated and unsaturated BMP accelerated protein aggregation. However, very little if any changes in the secondary structure of insulin fibrils grown in the presence of BMP were observed. Therefore, no changes in the toxicity of these aggregates compared to the fibrils formed in the lipid-free environment were observed. We also found that the toxicity of insulin oligomers formed in the presence of a 77:23 mol/mol ratio of BMP/PC, which represents the lipid composition of late endosomes, was slightly higher than the toxicity of insulin oligomers formed in the lipid-free environment. However, the toxicity of mature insulin fibrils formed in the presence of BMP/PC mixture was found to be lower or similar to the toxicity of insulin fibrils formed in the lipid-free environment. These results suggest that late endosomes are unlikely to be the source of highly toxic protein aggregates if amyloid proteins aggregate in them.

## Introduction

Amyloid oligomers and fibrils are highly toxic protein species formed as a result of abrupt aggregation of misfolded proteins.^1,2^ Fibrils typically possess several filaments that have a β-sheet secondary structure. These filaments can braid and intertwine, forming right- or left-twisted fibrils. Alternatively, fibril filaments can associate side-by-side, yielding tape-like constructs.^3−5^ Although the structural organization of amyloid fibrils is well understood from both solid-state NMR and cryo-EM studies,^6−9^ the secondary structure of amyloid oligomers remains unclear.^10−12^ Primarily due to the transient nature of these species and their high morphological heterogeneity that limit the use of solid-state NMR and cryo-EM for their structural characterization. This problem can be overcome with the use of optical nanoscopy techniques such as atomic force microscopy infrared (AFM-IR) spectroscopy.^13−17^ Zhou and Kurouski demonstrated that α-synuclein (α-Syn), a small protein that is directly linked to Parkinson’s disease, could form several different oligomers, from the perspective of their secondary structure, at the early and late stages of the protein aggregation.^18^ Some of these oligomers had primarily parallel β-sheet, whereas others possessed a mixture of β-sheet and α-helical protein.

Utilization of AFM-IR and other biophysical methods revealed that lipids can substantially alter the secondary structure of amyloid oligomers and fibrils.^16,17,19−24^ Furthermore, lipids were found to be present in protein aggregates formed in the presence of large unilamellar vesicles (LUVs).^13,16,17,20,25,26^ A growing body of evidence showed that such lipid-rich aggregates demonstrated drastically different cell toxicity compared to amyloid oligomers and fibrils formed in the lipid-free environment.^16,17,25,27−29^ It was also found that lipids not only changed the structure and toxicity of protein aggregates but also uniquely altered protein aggregation rates.^30−32^ Recently reported results by Zhaliazka and co-workers demonstrated that this effect is determined by the net charge of the lipid, degree of saturation, and length of fatty acids in these lipids.^27^

Matveyenka and co-workers found that insulin aggregates could be endocytosed by cells.^29^ This resulted in a degradation of endosomes and the leakage of fibrils into the cytosol, where these protein species were engaged in ROS production and mitochondrial damage.^17,26,28^ At the same time, numerous pieces of evidence suggest that protein aggregation can be triggered in cell organelles, such as endosomes and multivesicular bodies.^33−36^ For instance, Almeida and co-workers demonstrated that amyloid β peptide can accumulate in the multivesicular bodies, which results in fibril formation and cell death.^33^ Thus, endocytosis can cause an intake of fibrils that were previously formed in the extracellular space, as well as the accumulation of misfolded proteins that can aggregate in endosomes.^36^ In the latter case, the lipid composition of endosomes can play an important role in the aggregation of such proteins. The lipid profile of endosomes is dominated by bis(monoacylglycero)phosphate (BMP), an anionic lipid that is critically important for endosomal fusion.^37,38^ In late endosomes, BMP constitutes 77% of the total lipids.^39^ Expanding upon this, we investigate the role of this lipid, as well as the 77:23 mol/mol BMP/PC mixture, which represents the composition of late endosomes in insulin aggregation.^40,41^ Insulin aggregation is observed upon diabetes type 2 and injection amyloidosis.^42^ In a former case, the overproduction of insulin in the pancreas results in its aggregation. In the latter case, high local concentrations of insulin are created upon the hormone injection into the skin dermis.^43,44^ This not only leads to insulin aggregation but can also catalyze aggregation of other proteins present in cell media, which may result in systemic amyloidosis.^45^

Using a set of biophysical approaches, we determined the effect of BMP and BMP/PC on the rate of insulin aggregation. We also investigated the secondary structure and morphology of protein aggregates formed in the presence of these lipids. Finally, we utilized mice midbrain N27 cells to unravel the extent to which BMP and BMP/PC altered the toxicity of insulin fibrils.

## Results

### Kinetics of Insulin Aggregation

**Figure 1:**
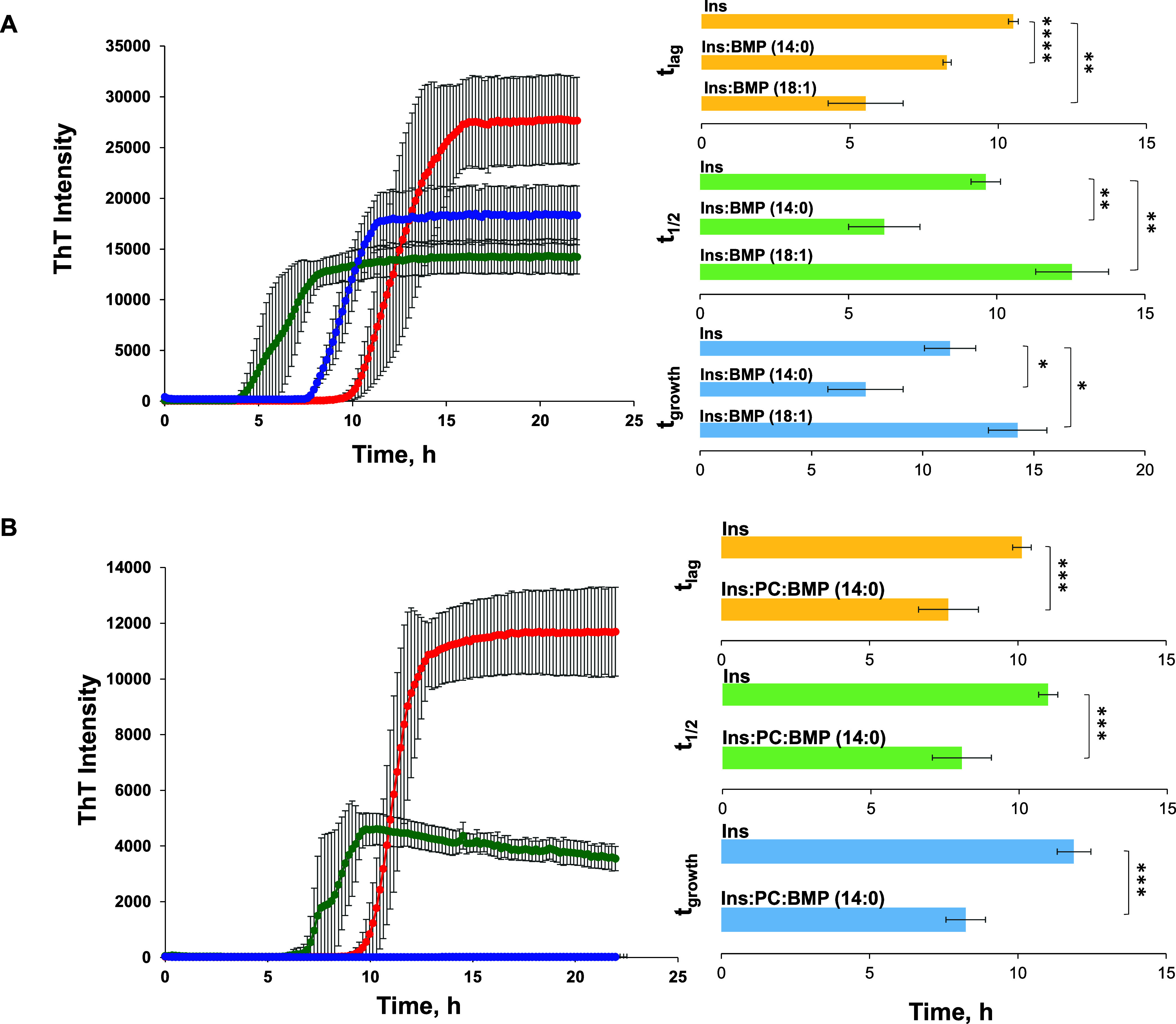
BMP and BMP/PC mixture uniquely alter the rate of insulin aggregation. ThT kinetics of insulin aggregation in the lipid-free environment (red) and in the presence of BMP(14:0) and BMP(18:1) at a 1:1 molar ratio (A) and in the presence of BMP(14:0):PC and BMP(18:1):PC in a 1:1 molar ratio (C). The corresponding values of *t*_lag_ (10% of max ThT intensity) and *t*_1/2_ (50% of max ThT intensity) and *t*_growth_ (90% of max ThT intensity) are shown in histograms (B) and (D), respectively. Each kinetic curve is the average of three independent measurements. All data were analyzed for normality using the Anderson–Darling test (*p* > 0.05). One-way ANOVA with Tukey HSD post-hoc test was used to determine significant differences between all samples reported in panel A (*p* < 0.05). The results of one-way ANOVA and the Tukey HSD post-hoc test are summarized in Tables S1–S3. The *T-*test was done to determine the statistical significance between the groups shown in the panel B. *P < 0.05; **P < 0.01; ***P < 0.001; ****P < 0.0001.

We employed a ThT assay to determine the effect of BMP on the rate of insulin aggregation. We found that both BMP(14:0) and BMP(18:1) drastically accelerated insulin aggregation if mixed in a 1:1 molar ratio with the protein. Specifically, we found that *t*_lag_ was shortened from 12.5 h (Ins) to 7.5 and 8.3 h in the case of BMP(14:0) and BMP(18:1), respectively, Figure 1. We also found that BMP altered the rate of insulin aggregation. Specifically, *t*_1/2_ changed from 16 h (Ins) to 8.2 and 11 h in the presence of BMP(14:0) and BMP(18:1), respectively. Furthermore, *t*_growth_ was found to be shortened from 21 h (Ins) to 12 and 13 h in the case of BMP(14:0) and BMP(18:1), respectively (Figure 1 and Tables S1–S3). These results are in good agreement with the previously reported findings by Matveyenka and co-workers. These results showed that anionic lipids such as PS and cardiolipin drastically accelerated the rate and significantly shortened the lag phase of insulin aggregation.^29^

Kinetic analysis of insulin aggregation of insulin aggregation in the presence of BMP/PC mixtures revealed that PC substantially decelerated the rate of insulin aggregation if mixed with BMP(14:0) and completely inhibited fibril formation if mixed with BMP(18:1) at a 23:77 PC:BMP mol/mol ratio. Specifically, we found that *t*_lag_ of insulin aggregation increased from 7.5 h (BMP(14:0)) to 8.5 h (BMP(14:0):PC), whereas *t*_1/2_ changed from 8.2 h (BMP(14:0)) to 10.3 h (BMP(14:0):PC) (Figure 1 and Tables S1–S3). These results demonstrate that the presence of zwitterionic lipids dramatically lowers the potential of anionic lipids in the acceleration of protein aggregation.^29^ Furthermore, our findings demonstrate that these effects are different for saturated and unsaturated BMPs.

### Morphological Analysis of Insulin Aggregates

**Figure 2:**
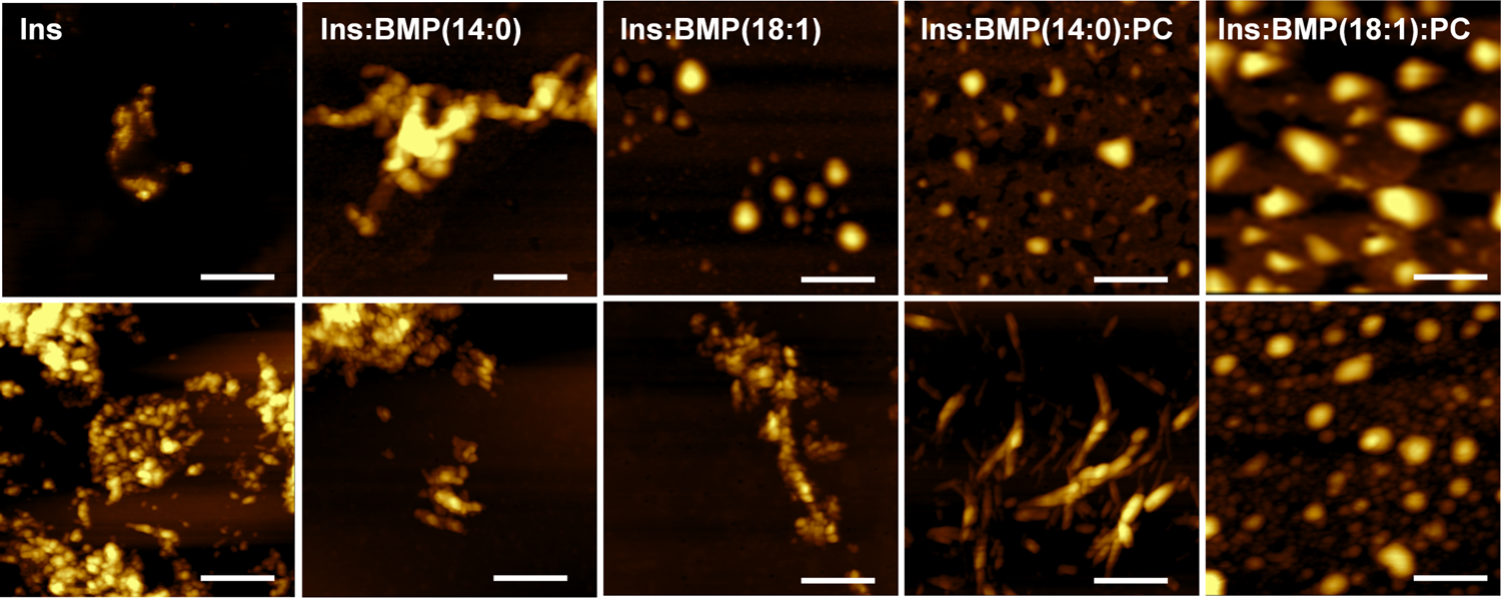
BMP and BMP/PC mixture uniquely alter the morphology of insulin aggregates. AFM images of insulin aggregates grown in the lipid-free environment (Ins), as well as in the presence of BMP(14:0) (Ins:BMP(14:0)), BMP(18:1) (Ins:BMP(18:1)), BMP(14:0):PC (Ins: BMP(14:0):PC), and BMP(18:1):PC (Ins:BMP(18:1):PC) at 5 h (top row) and 24 h (bottom row) of protein aggregation. The scale bar is 500 nm.

We used AFM to probe the topologies of insulin aggregates formed in the presence of BMP. AFM imaging revealed that at the early stage of insulin aggregation in the lipid-free environment, only small prefibrillar oligomers are formed (Figure 2). These aggregates later expanded into fibrillar structures that were abundant at 24 h of protein aggregation. We found that in the presence of BMP(14:0), insulin formed both oligomers are fibril-like structures already at 5 h of protein aggregation (Figure S1). Morphologically similar aggregates were found in Ins:BMP(14:0) at 24 h (Figure S1). However, we observed only small spherical protein aggregates present in Ins:BMP(18:1) at 5 h. In addition to these aggregates, we found LUVs that were ∼100 nm in diameter. However, in Ins:BMP(18:1) at 24 h, we observed protein aggregates and fibril-like species similar to those observed in Ins:BMP(14:0) at 24 h. These results demonstrate that both BMP(14:0) and BMP(18:1) alter the morphology of insulin oligomers formed at the early stage of protein aggregation.

Microscopic analysis of insulin aggregates formed at 5 h in the presence of Ins:BMP(14:0):PC and Ins:BMP(18:1):PC revealed the presence of small spherical protein aggregates together with LUVs (Ins:BMP(14:0):PC), whereas predominantly LUVs were observed in Ins:BMP(18:1):PC. We observed the formation of small protein aggregates in Ins:BMP(18:1):PC at 24 h. However, no fibril species were observed in this sample. At the same time, we found long fibrils in Ins:BMP(14:0):PC formed at 24 h. These results demonstrate that both BMP(14:0):PC and BMP(18:1):PC mixtures uniquely alter the morphology of insulin aggregates formed at 24 h in their presence.

### Structural Characterization of Protein Aggregates

**Figure 3:**
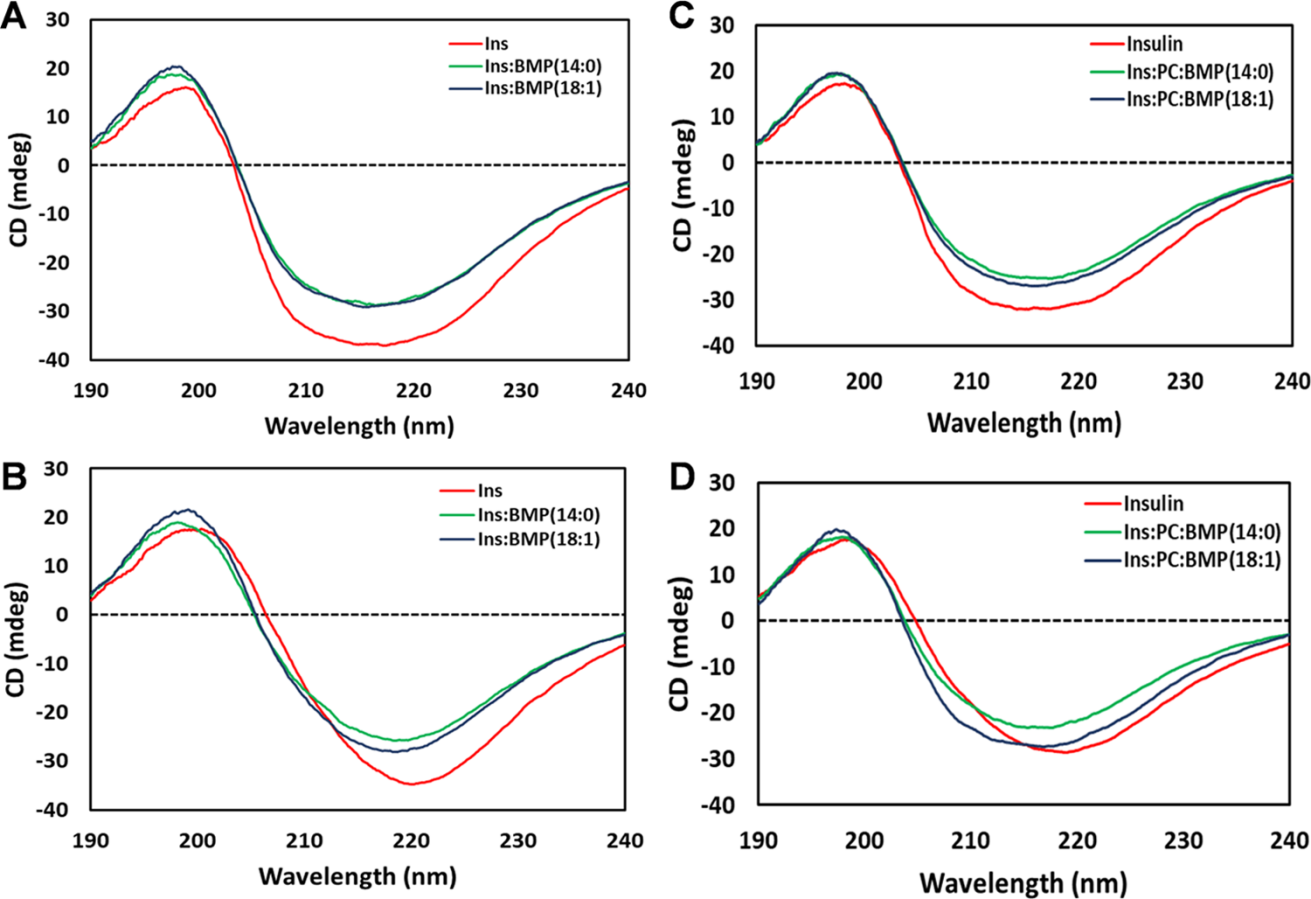
Structural analysis of insulin aggregates grown in the presence of BMP and BMP/PC mixture. CD spectra of insulin aggregates grown in the lipid-free environment (red), as well as in the presence of BMP(14:0) (green) and BMP(18:1) (blue) (A, B), as well as Ins:BMP(14:0):PC (green) and Ins:BMP(18:1):PC (blue) (C, D) after 5 h (A–C) and 24 h (B–D) of protein aggregation.

**Figure 4:**
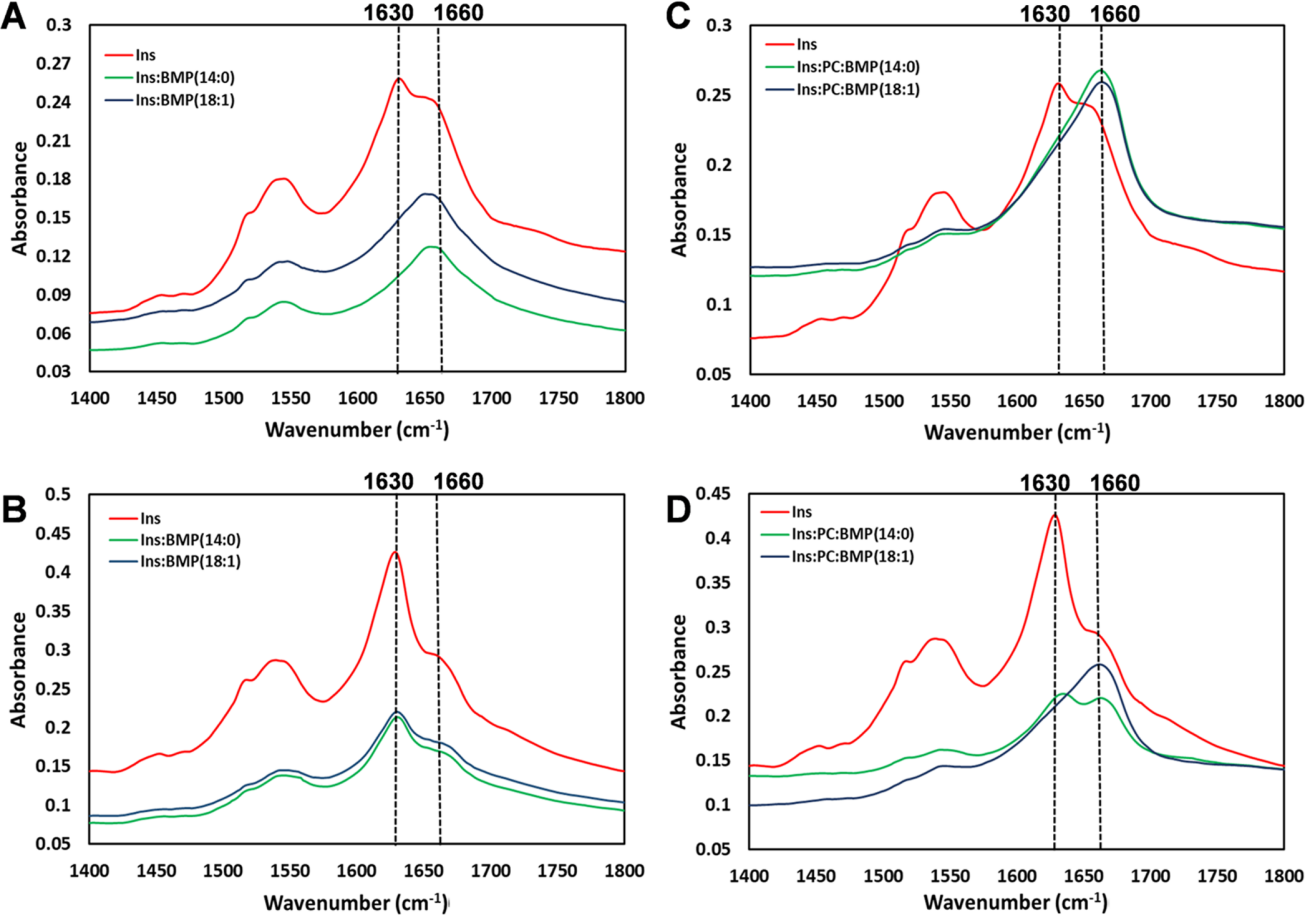
Structural analysis of insulin aggregates grown in the presence of BMP and BMP/PC mixture. FTIR spectra of insulin aggregates grown in the lipid-free environment (red) and in the presence of BMP(14:0) (green) and BMP(18:1) (blue) (A, B), as well as Ins:BMP(14:0):PC (green) and Ins:BMP(18:1):PC (blue) (C, D) after 5 h (A–C) and 24 h (B–D) of protein aggregation.

We utilized CD to examine the secondary structure of insulin aggregates grown in the presence of BMP and BMP/PC mixtures. We found that CD spectra acquired from a solution of insulin and Ins:BMP mixtures after 5 h of incubation at 37 °C had very similar CD spectra (Figure 3A,B). The same similarities were observed for the CD spectra acquired from insulin and Ins:BMP:PC after 5 h of initiation of protein aggregation (Figure 4). These spectra exhibited a broad trough with maxima around 217 nm, which indicates the presence of a mixture of unordered protein, α-helix, and β-sheet secondary structures in the analyzed protein samples (Table S4). One can expect that unordered protein and α-helix originate from partially native insulin that was not able to aggregate at this time point, whereas the β-sheet secondary structure originates from protein oligomers.

**Figure 5:**
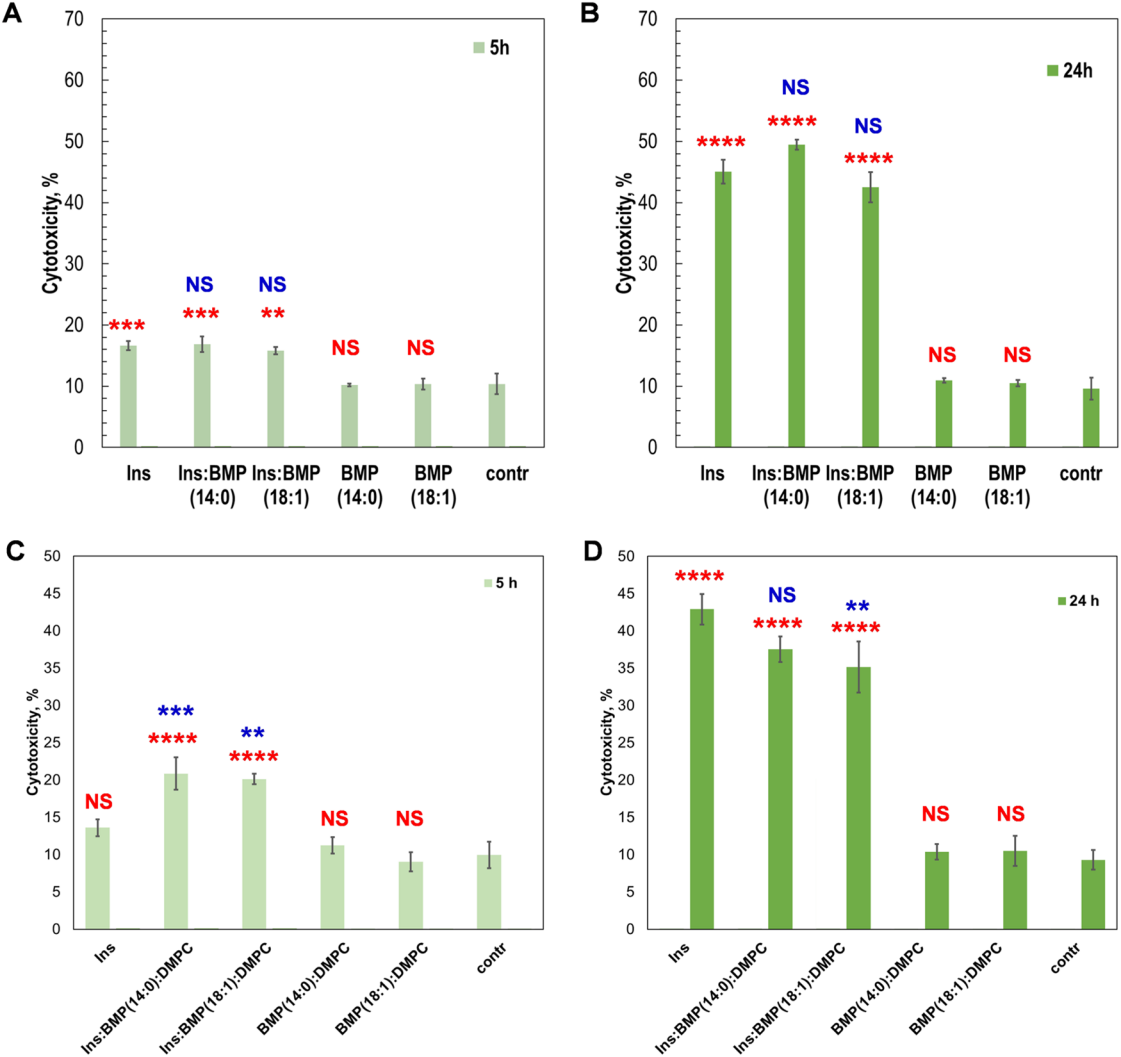
Insulin aggregates grown in the presence of BMP exhibit similar cell toxicity compared to the aggregates grown in the lipid-free environment, whereas BMP/PC lowered the toxicity of insulin aggregates. Histograms of LDH assay of Ins, Ins:BMP(14:0), and Ins:BMP(18:1), as well as BMP(14:0) and BMP(18:1) themselves on day 5 (A) and day 24 (B) after the initiation of protein aggregation. Histograms of LDH assay of LDH assay of Ins, Ins:BMP(14:0):PC, and Ins:BMP(18:1):PC, as well as BMP(14:0):PC and BMP(18:1):PC formed on day 5 (C) and day 24 (D) after the initiation of protein aggregation. Measurements were made in triplicates. Data were analyzed for normality using the Anderson–Darling test (*p* > 0.05). One-way ANOVA showed significant differences between samples (*p* < 0.05), and Tukey HSD post-hoc test was used for further group comparison (Tables S5 and S6). Red asterisks (*) show the statistical significance of all samples compared to the control. Blue asterisks show statistical significance between Ins and insulin aggregates formed in the presence of BMP. *P < 0.05; **P < 0.01; ***P < 0.001; ****P < 0.0001; NS, nonsignificant difference.

The CD spectra of insulin and Ins:BMP mixtures incubated for 24 h changed significantly compared to the CD spectra of these samples acquired after 5 h of incubation (Figure 3C,D). Specifically, we found that spectral maxima red shifted to ∼220 nm, which indicates the predominance of the β-sheet secondary structure. At the same time, we found that the CD spectrum of insulin had a maximum at 220 nm, whereas the CD spectra of both insulin:14:0:BMP and insulin:18:1:BMP mixtures exhibited this maximum at ∼217 nm (Table S1). Similar changes in CD spectra were observed for Ins/BMP/PC mixtures at 24 h (Figure 5). Specifically, the CD spectra of both Ins:BMP(14:0):PC and Ins:BMP(18:1):PC mixtures exhibited this maximum at ∼217 nm. Thus, one can expect small differences in the secondary structure of insulin aggregates grown in the lipid-free environment and in the presence of BMP and a BMP/PC mixture.

The ATR-FTIR spectra acquired from insulin aggregates formed at 5 h after the initiation of protein aggregation exhibit two vibrational bands known as amide I (1500–1560 cm^–1^) and amide I (1600–1700 cm^–1^). Amide I band exhibits a maximum at ∼1630 cm^–1^, which indicates the presence of the β-sheet secondary structure in the insulin aggregates. We also observed a shoulder at ∼1660 cm^–1^ in the ATR-FTIR spectrum of insulin aggregates, which points to the substantial amount of unordered protein in the analyzed sample. At the same time, the ATR-FTIR spectra acquired from both 14:0 and 18:1 Ins:BMP aggregates, as well as from Ins:BMP:PC aggregates formed at 5 h after the initiation of protein aggregation, exhibit a peak centered at ∼1660 cm^–1^, which suggests about the predominance of unordered protein in these samples (Figure 4A,B).

Ins and Ins:BMP aggregates formed at 24 h after the initiation of protein aggregation exhibit very similar if not identical spectra. We found an intense amide I centered at ∼1630 cm^–1^ with a shoulder at ∼1660 cm^–1^ in all acquired ATR-FTIR spectra, which suggests the predominance of a parallel β-sheet with some amount of unordered protein secondary structure in these aggregates (Figure 4C,D). At the same time, Ins:BMP(18:1):PC aggregates formed at 24 h exhibited an IR spectrum with only intense vibration at ∼1660 cm^–1^, which indicates the predominance of unordered protein secondary structure in these aggregates. However, the IR spectrum acquired from Ins:BMP(14:0):PC aggregates formed at 24 h was found to be very similar to the IR spectrum of Ins:BMP(14:0) aggregates, indicating the predominance of parallel β-sheet with some amount of unordered protein secondary structure in these aggregates (Figure 4C,D).

### Toxicity of Insulin Aggregates

The question to ask is whether insulin aggregates grown in the presence of BMP exert different cell toxicity. To answer this question, we investigate the extent to which Ins, Ins:BMP(14:0), and Ins:BMP(18:1) aggregates exert cell toxicity and cause ROS stress to mice midbrain N27 cell line (Figure 5). We found that the presence of BMP alone did not significantly alter the toxicity of insulin aggregates formed at both 5 and 24 h compared to the toxicity of insulin oligomers and fibrils grown in the lipid-free environment. However, it should be noted that Ins and Ins:BMP(14:0 and 18:1) aggregates exerted statistically significant cell toxicity, whereas lipids themselves were found to be insignificantly toxic to N27 cells.

We also found that insulin oligomers grown at 5 h of insulin aggregation in the presence of BMP/PC exerted slightly higher cell toxicity compared to insulin oligomers grown in the lipid-free environment. However, Ins:BMP(18:1):PC fibrils formed at 24 h exerted significantly lower cell toxicity compared to the insulin fibrils formed in the lipid-free environment (Figure 5). Finally, we did not find significant differences between the toxicity of Ins and Ins:BMP(14:0):PC fibrils. These results demonstrate that insulin oligomers that are formed at the early state of aggregation in the presence of BMP/PC mixture appear slightly more toxic than corresponding oligomers grown in the lipid-free environment. Our previously reported results demonstrated that PC strongly inhibited insulin aggregation. Therefore, one can envision that the presence of PC is the underlying molecular cause of the observed decrease in the toxicity of Ins:BMP(18:1):PC fibrils. However, the observed difference between the toxicity of Ins:BMP(14:0):PC and Ins:BMP(18:1):PC fibrils suggested that the degree of saturation of BMP determines the toxicity of insulin aggregates formed in the presence of BMP/PC mixture.

## Discussion

Our previously reported findings demonstrated that anionic lipids strongly accelerated protein aggregation. Experimental results reported in this work confirm these findings. We found that the presence of BMP at a 1:1 molar ratio with insulin significantly accelerated fibril formation compared to the rate of insulin aggregation in the lipid-free environment. We also found that the presence of PC at 23 to 77% of BMP in LUVs had no effect on the effect of BMP(14:0) exerted on the rate of insulin aggregation. However, the presence of PC in LUVs fully canceled the effects exerted by BMP(18:1) that were previously seen. Specifically, we observed no protein aggregation in the presence BMP(18:1):PC. Thus, saturation and the length of fatty acids in BMP play an important role in protein aggregation when the lipid is mixed with PC.

We also found that BMP itself did not alter the secondary structure and toxicity of early-stage oligomers formed in its presence. However, we observed some changes in the secondary structure of Ins:BMP fibrils formed at 24 h. Nevertheless, there was no substantial difference in the toxicity of such fibrils compared to the insulin aggregates grown in a lipid-free environment. Our findings show that lipid mixtures exert different effects on the aggregation of proteins compared to mono lipids. Specifically, we found that early-stage oligomers formed in the presence of both BMP(14:0) and BMP(18:1) exerted slightly higher cell toxicity compared to insulin aggregates grown in a lipid-free environment. However, the toxicity of mature Ins:BMP(18:1):PC fibrils was found to be lower than the toxicity of Ins fibrils. These results suggest that late endosomes, even with 77% BMP present in them, were unlikely to generate highly toxic fibrils compared to the protein aggregates that could be formed on the surface of plasma membrane that contains PS or in mitochondria that possesses cardiolipin.

## Methods

### Materials

Bovine insulin was purchased from Sigma-Aldrich (St. Louis, MO), and 14:0 and 18:1 BMPs were purchased from Avanti (Alabaster, AL).

### Liposome Preparation

BMP and PC dissolved in ethanol were first mixed in a 77:23 mol/mol ratio and dried at room temperature. Next, 0.6 mg of BMP or BMP/PC mixture was dissolved in 2.6 mL of phosphate-buffered saline (PBS) of pH 7.4. A vial containing lipid solutions was heated in a water bath to ∼50 °C for 30 min. Next, the vial was placed into liquid nitrogen for 3–5 min. This procedure was repeated 10 times. After this, solutions of lipids were passed 15 times through a 100 nm membrane that was placed into the extruder (Avanti, Alabaster, AL). LUV sizes were determined by dynamic light scattering.

### Insulin Aggregation

In a lipid-free environment, 400 μM of insulin was dissolved in PBS. The solution pH was adjusted to 3.0 using concentrated HCl. For Ins:BMP and Ins:BMP:PC, 400 μM of insulin was mixed with an equivalent concentration of the corresponding lipid, and the solution pH was adjusted to 3.0 using concentrated HCl. Next, the solutions were placed in a plate reader (Tecan, Männedorf, Switzerland) and incubated at 37 °C under 510 rpm for 24 h.

### Kinetic Measurements

Insulin aggregation was monitored using a thioflavin T (ThT) fluorescence assay. Briefly, protein samples were mixed with 2 mM of the ThT solution and placed in a plate reader (Tecan, Männedorf, Switzerland) where the samples were incubated at 37 °C under 510 rpm for 30 h. Fluorescence measurements were taken every 10 min. Three independent experiments were made for each of the reported results. All data were analyzed for normality using the Anderson–Darling test (*p* > 0.05). One-way ANOVA with Tukey HSD post-hoc test was used to determine significant differences between all samples reported in panel A (Figure 1; *p* < 0.05). The results of one-way ANOVA and the Tukey HSD post-hoc test are summarized in Tables S1–S3. The *T-*test was done to determine the statistical significance between the groups shown in panel B (Figure 1; *P < 0.05; **P < 0.01; ***P < 0.001; ****P < 0.0001).

### AFM Imaging

AFM imaging was performed using silicon AFM probes with related parameters force constant 2.7 N/m and resonance frequency 50–80 kHz were purchased from Appnano (Mountain View, CA) on AIST-NT-HORIBA system (Edison, NJ). An analysis of the collected images was performed using AIST-NT software (Edison, NJ). For each sample, an aliquot of protein aggregates was diluted with DI water and deposited onto a glass cover slide. Next, two to three sample areas were analyzed using AFM to ensure that reported AFM images were representative of the analyzed samples.

### Circular Dichroism (CD)

After 5 and 24 h of sample incubation, the samples were diluted to the final concentration of 100 μM using PBS and measured immediately using a J-1000 CD spectrometer (Jasco, Easton, MD). Three spectra were collected for each sample within 205–250 nm.

### Attenuated Total Reflectance Fourier Transform Infrared (ATR-FTIR) Spectroscopy

After 5 and 24 h of sample incubation, the samples were placed onto an ATR crystal and dried at room temperature. The spectra were measured using a Spectrum 100 FTIR spectrometer (Perkin-Elmer, Waltham, MA). Three spectra were collected from each sample.

### Cell Toxicity Assays

Rat midbrain N27 cells were grown in RPMI 1640 medium (Thermo Fisher Scientific, Waltham, MA) with 10% fetal bovine serum (FBS) (Invitrogen, Waltham, MA) in a 96-well plate (10,000 cells per well) at 37 °C under 5% CO_2_. After 24 h, the cells were found to fully adhere to the wells, reaching ∼70% confluency. Next, 100 μL of the cell culture was replaced with 100 μL of the RPMI 1640 medium with 5% FBS-containing protein samples. After 24 h of incubation, lactate dehydrogenase (LDH) assay was performed on the cell medium using CytoTox 96 nonradioactive cytotoxicity assay (G1781, Promega, Madison, WI). Absorption measurements were made in a plate reader (Tecan, Männedorf, Switzerland) at 490 nm. All measurements were made in triplicates.

## Conclusions

Our results demonstrated that although BMP itself accelerated theprotein aggregation, no significant changes was observed in the toxicity of insulin aggregates formed in the presence of this lipid. We also found that a small concentration of PC relative to BMP (23:77 mol/mol ratio) changed the effect of BMP(18:1) on the rate of insulin aggregation. However, very little if any changes were observed for BMP(14:0). We also found that oligomers formed at the early stage of insulin aggregation in the presence of BMP(14:0):PC and BMP(18:1):PC exerted slightly higher cell toxicity compared to the oligomers formed by insulin in the lipid-free environment. However, the toxicity of mature Ins:BMP(18:1):PC fibrils formed at 24 h of protein aggregation was found to be lower than the toxicity of both Ins and Ins:BMP(14:0):PC fibrils. These results suggest that BMP and late endosomes are unlikely the source of high-toxicity protein aggregates. Therefore, it is highly likely that the plasma, rather than late endosome membranes, play a key role in developing highly toxic protein oligomers and fibrils that contribute to the onset and spread of neurodegenerative diseases.

## Supporting Information Available

Height profiles for protein aggregates observed by AFM are reported in Figure S1. The secondary structure of protein aggregates according to the deconvolution of CD spectra reported in Figure 2 are summarized in Table S1 (PDF)
