# Supplementary material for: Elucidation of the Role of Lipids in Late Endosomes on the Aggregation of Insulin
Source: ACS Chem Neurosci. 2023 Sep 8;14(18):3551–9. doi: 10.1021/acschemneuro.3c00475 (PMC10862470; doi:10.1021/acschemneuro.3c00475)
Supplement: Supplementary file 1 — cn3c00475_si_001.pdf [file cn3c00475_si_001.pdf]

Elucidation of the Role of Lipids in Late Endosomes on the Aggregation of Insulin

Ritu Joshi[1], Kiryl Zhaliazka[1], Aidan Holman[2], and Dmitry Kurouski[*1,3]

1. Department of Biochemistry and Biophysics, Texas A&M University, College Station, Texas 77843, United States
2. Department of Entomology, Texas A&M University, College Station, Texas 77843, United States
3. Department of Biomedical Engineering, Texas A&M University, College Station, Texas, 77843, United States

Correspondence: dkurouski@tamu.edu

Supporting Information

Table S1. For t_{lag} data was analyzed for normality using the Anderson-Darling Test ($p > 0.05$). One-Way ANOVA showed significant differences between samples ($p < 0.05$), and the Tukey HSD posthoc test was used for further group comparison.

Group A	Group B	Lower Limit	A-B	Upper Limit	P-value	
Ins:BMP(14:0)	Ins:BMP(18:1)	-4.208248557	-2.744346528	-1.280444499	0.001399407	**
Ins:BMP(14:0)	Ins	-6.445933002	-4.982030973	-3.518128944	1.45925E-05	****
Ins:BMP(18:1)	Ins	-3.701586474	-2.237684445	-0.773782416	0.005315625	**

Table S2. For $t_{l/2}$ data was analyzed for normality using the Anderson-Darling Test ($p > 0.05$). One-Way ANOVA showed significant differences between samples ($p < 0.05$), and the Tukey HSD posthoc test was used for further group comparison.

Group A	Group B	Lower Limit	A-B	Upper Limit	P-value	
Ins:BMP(14:0)	Ins:BMP(18:1)	4.291318315	6.33305736	8.37479641	3.11228E-05	****
Ins:BMP(14:0)	Ins	0.871449496	2.91318854	4.95492759	0.008053718	**
Ins:BMP(18:1)	Ins	-5.461607869	-3.41986882	-1.37812977	0.002976968	**

Table S3. For t_{growth} data was analyzed for normality using the Anderson-Darling Test ($p > 0.05$). One-Way ANOVA showed significant differences between samples ($p < 0.05$), and the Tukey HSD posthoc test was used for further group comparison.

Group A	Group B	Lower Limit	A-B	Upper Limit	P-value	
Ins:BMP(14:0)	Ins:BMP(18:1)	4.067929142	6.83970382	9.6114785	0.000188806	***
Ins:BMP(14:0)	Ins	0.268089904	3.03986458	5.81163926	0.032817843	*
Ins:BMP(18:1)	Ins	-6.571613917	-3.79983924	-1.02806456	0.01016038	*

Table S4. The secondary structure of protein aggregates according to the deconvolution of CD spectra reported in Figure 2.

Secondary Structure	α-helix	β-sheet	Random coil
Ins 5h	25	52	23
Ins:BMP (14:0) 5h	22	57	21
Ins:BMP (18:1) 5h	22	57	21
Ins:PC:BMP (14:0) 5h	22	57	21
Ins:PC:BMP (18:1) 5h	22	57	21
Ins 24h	9.5	75.5	17
Ins:BMP (14:0) 24h	13	73	14
Ins:BMP (18:1) 24h	12	74	14
Ins:PC:BMP (14:0) 5h	13	72	15
Ins:PC:BMP (18:1) 5h	13	72	15

Table S5. One-way ANOVA shows Significant differences for all testing groups. Tukey's HSD Post Hoc was performed for multiple comparison procedures and the statistical test, showed the following difference between tested groups:

	Group A	Group B	Lower Limit	A-B	Upper Limit	P-value	
5 h	Ins	Ins:BMP(14:0):DMPC	-11.221	-7.267	-3.312	0.000527853	***

	Group A	Group B	Lower Limit	A-B	Upper Limit	P-value	
5 h	Ins	Ins:BMP(14:0)	-0.04	0.00	0.03	9.999E-01	NS
	Ins	Ins:BMP(18:1)	-0.03	0.01	0.04	9.566E-01	NS
	Ins	BMP(14:0)	0.03	0.06	0.10	3.875E-04	***
	Ins	BMP(18:1)	0.03	0.06	0.10	4.935E-04	***
	Ins	contr	0.03	0.06	0.10	4.935E-04	***
	Ins:BMP(14:0)	Ins:BMP(18:1)	-0.02	0.01	0.04	8.886E-01	NS
	Ins:BMP(14:0)	BMP(14:0)	0.03	0.07	0.10	2.778E-04	***
	Ins:BMP(14:0)	BMP(18:1)	0.03	0.07	0.10	3.522E-04	***
	Ins:BMP(14:0)	contr	0.03	0.07	0.10	3.522E-04	***
	Ins:BMP(18:1)	BMP(14:0)	0.02	0.06	0.09	1.342E-03	**
	Ins:BMP(18:1)	BMP(18:1)	0.02	0.05	0.09	1.737E-03	**
	Ins:BMP(18:1)	contr	0.02	0.05	0.09	1.737E-03	**
	BMP(14:0)	BMP(18:1)	-0.04	0.00	0.03	1.000E+00	NS
	BMP(14:0)	contr	-0.04	0.00	0.03	1.000E+00	NS
	BMP(18:1)	contr	-0.03	0.00	0.03	1.000E+00	NS
24h	Ins	Ins:BMP(14:0)	-0.04	-0.04	0.01	1.095E-01	NS
	Ins	Ins:BMP(18:1)	-0.03	0.03	0.08	5.667E-01	NS
	Ins	BMP(14:0)	0.03	0.34	0.39	3.167E-10	****
	Ins	BMP(18:1)	0.03	0.35	0.40	2.649E-10	****
	Ins	contr	0.03	0.35	0.41	1.828E-10	****
	Ins:BMP(14:0)	Ins:BMP(18:1)	-0.02	0.07	0.12	6.598E-03	**
	Ins:BMP(14:0)	BMP(14:0)	0.03	0.39	0.44	5.142E-11	****
	Ins:BMP(14:0)	BMP(18:1)	0.03	0.39	0.44	4.292E-11	****
	Ins:BMP(14:0)	contr	0.03	0.40	0.45	2.944E-11	****
	Ins:BMP(18:1)	BMP(14:0)	0.02	0.32	0.37	9.181E-10	****
	Ins:BMP(18:1)	BMP(18:1)	0.02	0.32	0.37	7.662E-10	****
	Ins:BMP(18:1)	contr	0.02	0.33	0.38	5.273E-10	****
	BMP(14:0)	BMP(18:1)	-0.04	0.00	0.06	9.997E-01	NS
	BMP(14:0)	contr	-0.04	0.01	0.06	9.460E-01	NS
	BMP(18:1)	contr	-0.03	0.01	0.06	9.898E-01	NS

Table S6. One-way ANOVA shows Significant differences for all testing groups. Tukey's HSD Post Hoc was performed for multiple comparison procedures and the statistical test, showed the following difference between tested groups:

	Ins	Ins:BMP(18:1):DMPC	-10.488	-6.533	-2.579	0.001356318	**
	Ins	BMP(14:0):DMPC	-1.621	2.333	6.288	0.40451133	
	Ins	BMP(18:1):DMPC	0.612	4.567	8.521	0.020815269	*
	Ins	contr	-0.321	3.633	7.588	0.078683879	NS
	Ins:BMP(14:0):DMPC	Ins:BMP(18:1):DMPC	-3.221	0.733	4.688	0.987026088	NS
	Ins:BMP(14:0):DMPC	BMP(14:0):DMPC	5.645	9.600	13.555	3.56546E-05	****
	Ins:BMP(14:0):DMPC	BMP(18:1):DMPC	7.879	11.833	15.788	3.98923E-06	****
	Ins:BMP(14:0):DMPC	contr	6.945	10.900	14.855	9.56277E-06	****
	Ins:BMP(18:1):DMPC	BMP(14:0):DMPC	4.912	8.867	12.821	7.92118E-05	****
	Ins:BMP(18:1):DMPC	BMP(18:1):DMPC	7.145	11.100	15.055	7.89188E-06	****
	Ins:BMP(18:1):DMPC	contr	6.212	10.167	14.121	1.9793E-05	****
	BMP(14:0):DMPC	BMP(18:1):DMPC	-1.721	2.233	6.188	0.44796738	NS
	BMP(14:0):DMPC	contr	-2.655	1.300	5.255	0.870627386	NS
	BMP(18:1):DMPC	contr	-4.888	-0.933	3.021	0.963503331	NS
24 h	Ins	Ins:BMP(14:0):DMPC	-0.297	5.367	11.030	0.067045461	NS
	Ins	Ins:BMP(18:1):DMPC	2.070	7.733	13.397	0.006357633	**
	Ins	BMP(14:0):DMPC	26.836	32.500	38.164	2.27374E-09	****
	Ins	BMP(18:1):DMPC	26.736	32.400	38.064	2.36379E-09	****
	Ins	contr	27.936	33.600	39.264	1.48802E-09	****
	Ins:BMP(14:0):DMPC	Ins:BMP(18:1):DMPC	-3.297	2.367	8.030	0.724739173	NS
	Ins:BMP(14:0):DMPC	BMP(14:0):DMPC	21.470	27.133	32.797	2.02638E-08	****
	Ins:BMP(14:0):DMPC	BMP(18:1):DMPC	21.370	27.033	32.697	2.11614E-08	****
	Ins:BMP(14:0):DMPC	contr	22.570	28.233	33.897	1.26731E-08	****
	Ins:BMP(18:1):DMPC	BMP(14:0):DMPC	19.103	24.767	30.430	5.85031E-08	****
	Ins:BMP(18:1):DMPC	BMP(18:1):DMPC	19.003	24.667	30.330	6.1289E-08	****
	Ins:BMP(18:1):DMPC	Contr	20.203	25.867	31.530	3.54089E-08	****
	BMP(14:0):DMPC	BMP(18:1):DMPC	-5.764	-0.100	5.564	0.999999842	NS
	BMP(14:0):DMPC	contr	-4.564	1.100	6.764	0.984083733	NS
	BMP(18:1):DMPC	contr	-4.464	1.200	6.864	0.976797978	NS

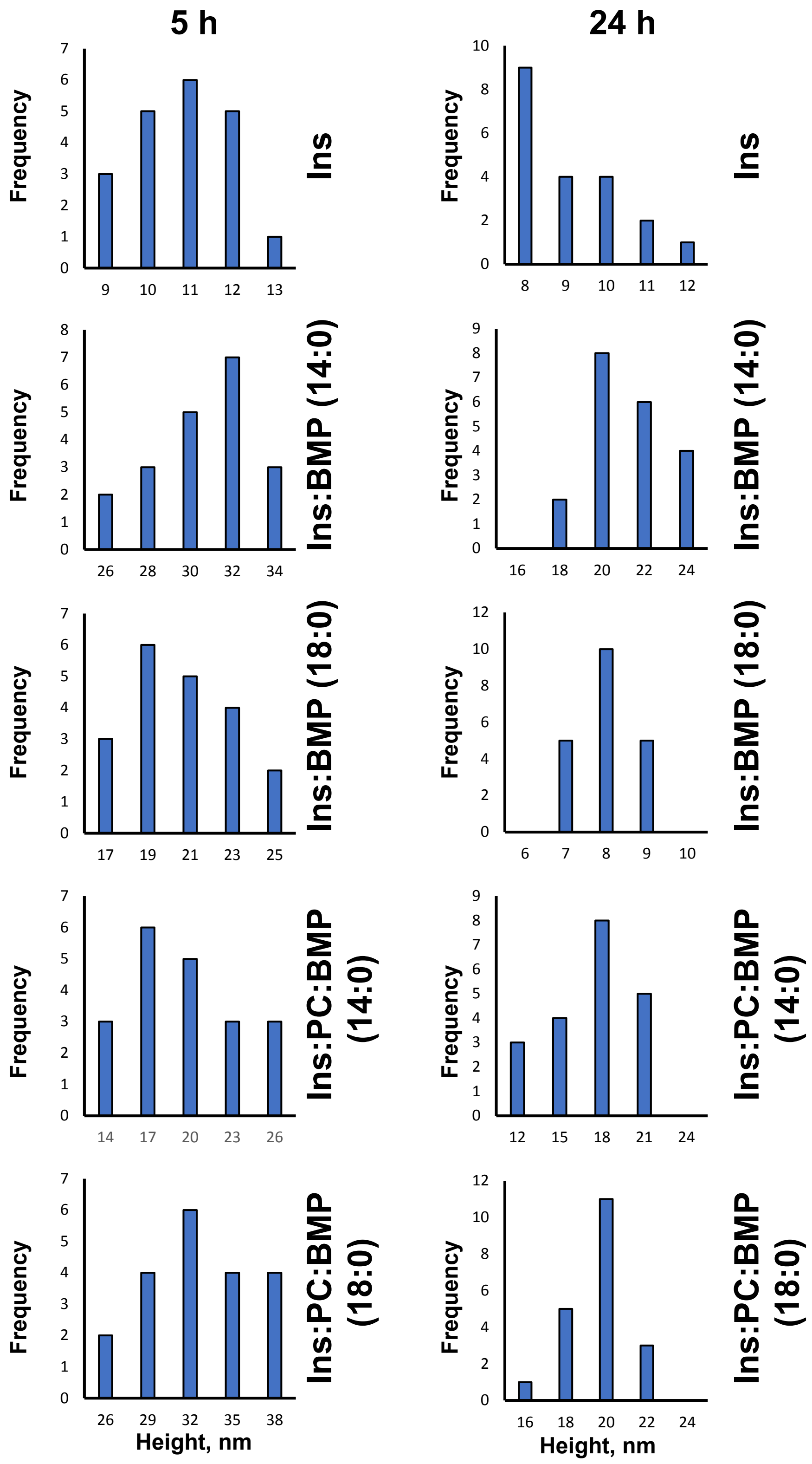

Figure S1. Height profiles of protein aggregates.
